# Influence of field of view size on image quality: ultra-high-resolution CT vs. conventional high-resolution CT

**DOI:** 10.1007/s00330-020-06704-0

**Published:** 2020-02-18

**Authors:** Tomo Miyata, Masahiro Yanagawa, Akinori Hata, Osamu Honda, Yuriko Yoshida, Noriko Kikuchi, Mitsuko Tsubamoto, Shinsuke Tsukagoshi, Ayumi Uranishi, Noriyuki Tomiyama

**Affiliations:** 1 Department of Radiology, Osaka University Graduate School of Medicine, 2-2 Yamadaoka, Suita City, Osaka 565-0871 Japan; 2 Department of Future Diagnostic Radiology, Osaka University Graduate School of Medicine, 2-2 Yamadaoka, Suita City, Osaka 565-0871 Japan; 3 Department of CT Systems, Canon Medical Systems Corp., Otawara, Tochigi Japan

**Keywords:** Image enhancement, Diagnostic imaging, Lung diseases

## Abstract

**Objectives:**

This study was conducted in order to compare the effect of field of view (FOV) size on image quality between ultra-high-resolution CT (U-HRCT) and conventional high-resolution CT (HRCT).

**Methods:**

Eleven cadaveric lungs were scanned with U-HRCT and conventional HRCT and reconstructed with five FOVs (40, 80, 160, 240, and 320 mm). Three radiologists evaluated and scored the images. Three image evaluations were performed, comparing the image quality with the five FOVs with respect to the 160-mm FOV. The first evaluation was performed on conventional HRCT images, and the second evaluation on U-HRCT images. Images were scored on normal structure, abnormal findings, and overall image quality. The third evaluation was a comparison of the images obtained with conventional HRCT and U-HRCT, with scoring performed on overall image quality. Quantitative evaluation of noise was performed by setting ROIs.

**Results:**

In conventional HRCT, image quality was improved when the FOV was reduced to 160 mm. In U-HRCT, image quality, except for noise, improved when the FOV was reduced to 80 mm. In the third evaluation, overall image quality was improved in U-HRCT over conventional HRCT at all FOVs. Noise of U-HRCT increased with respect to conventional HRCT when the FOV was reduced from 160 to 40 mm. However, at 240- and 320-mm FOVs, the noise of U-HRCT and conventional HRCT showed no differences.

**Conclusions:**

In conventional HRCT, image quality did not improve when the FOV was reduced below 160 mm. However, in U-HRCT, image quality improved even when the FOV was reduced to 80 mm.

**Key Points:**

*• Reducing the size of the field of view to 160 mm improves diagnostic imaging quality in high-resolution CT.*

*• In ultra-high-resolution CT, improvements in image quality can be obtained by reducing the size of the field of view to 80 mm.*

*• Ultra-high-resolution CT produces images of higher quality compared with conventional HRCT irrespective of the size of the field of view.*

**Electronic supplementary material:**

The online version of this article (10.1007/s00330-020-06704-0) contains supplementary material, which is available to authorized users.

## Introduction

Smaller fields of views (FOVs) in CT are generally associated with higher spatial resolution and clearer images. As the image quality becomes clearer, the ability to find lung nodules and the diagnostic ability to distinguish between benign and malignant nodules are improved [1, 2]. However, noise is increased and image quality deteriorated by reducing the FOV [3, 4]. Recently, ultra-high-resolution CT (U-HRCT) has been developed, characterized by a smaller focal size of the detector elements and the X-ray tube compared with conventional high-resolution CT (conventional HRCT), allowing clearer CT images [5]. U-HRCT with 1024 × 1024 matrix size has been reported to improve image quality and diagnostic potential [6], and that a U-HRCT matrix size of 2048 × 2048 leads to larger improvement than sizes 512 × 512 or 1024 × 1024 [7]. However, no studies investigated the influence of FOV size on image quality in U-HRCT. The spatial resolution of conventional HRCT has been reported to range from 0.23 to 0.35 mm, and that of U-HRCT to be 0.14 mm [5, 7–9]. One pixel of matrix size (512 × 512) corresponds to 0.156 mm (0.078 mm) when the FOV size is 80 mm (40 mm). If the pixel size at a given FOV is smaller than the spatial resolution of conventional HRCT, the image quality cannot improve even if the FOV is further decreased. Therefore, we hypothesized that even at FOV sizes for which the image quality is not further improved in conventional HRCT, an improvement could still be seen in U-HRCT. The purpose of our study was to compare the effect of FOV size on image quality in U-HRCT and conventional HRCT, so as to test such hypothesis.

## Materials and methods

### Cadaveric lungs

The study was approved by our Institutional Review Board and did not require informed consent due to its retrospective design. Eleven cadaveric lungs from patients with a history of pulmonary disease were used. The lungs were inflated and fixed using Heitzman’s method [10], namely distended through the main bronchus with fixative fluid containing polyethylene glycol 400, 95% ethyl alcohol, 40% formalin, and water in the proportion of 10:5:2:3. The specimens were immersed in the fixative fluid for 2 days and then air-dried.

These samples were pathologically characterized as tuberculosis (*n* = 2), usual interstitial pneumonia (*n* = 2), diffuse pan bronchiolitis, metastatic disease, cardiogenic edema, diffuse alveolar hemorrhage, diffuse alveolar damage, emphysema, and lymphangitic carcinomatosis (*n* = 1 each).

### U-HRCT and conventional HRCT scanners

U-HRCT (Aquilion Precision™; Canon Medical Systems) and conventional HRCT (Aquilion ONE™; Canon Medical Systems) were used as CT scanners. In U-HRCT, the detector element size is 0.25 × 0.25 mm, with 160 rows and 1792 channels, and the focus size of the X-ray tube is 0.4 × 0.5 mm. On the other hand, in conventional HRCT, the detector element size is 0.5 × 0.5 mm, with 320 rows and 896 channels, and the focus size of the X-ray tube is 0.8 × 0.9 mm. The detector size of the U-HRCT scanner is thus half the size of the conventional CT detector elements in both the *x*–*y* plane and the *z*-axis direction, and the focus size of the X-ray tube in U-HRCT is approximately 1/3 that of conventional HRCT.

### Image acquisition

**Fig. 1:**
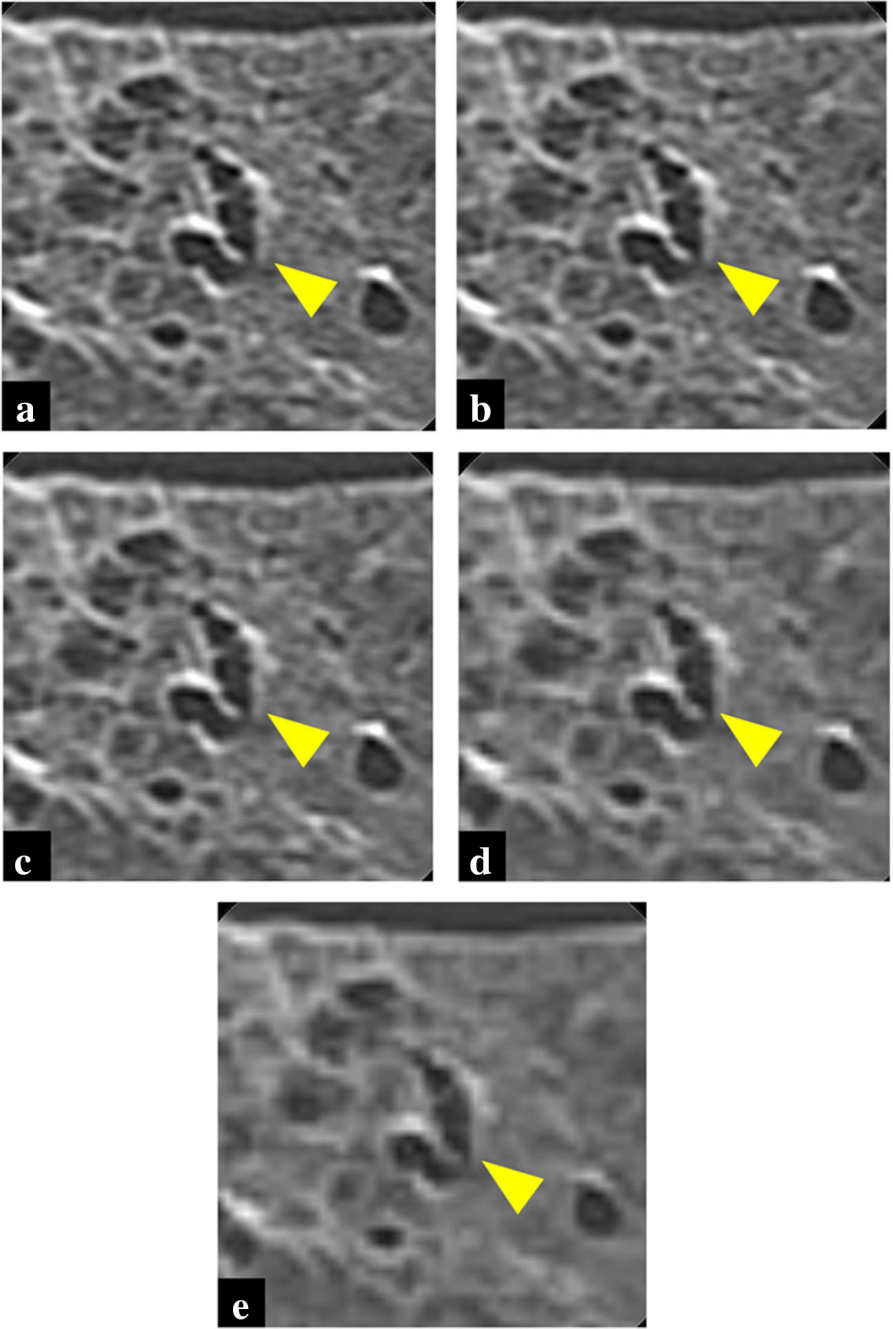
Images of conventional HRCT of cadaveric lung (usual interstitial pneumonia) at FOV 40 mm (**a**), 80 mm (**b**), 160 mm (**c**), 240 mm (**d**), and 320 mm (**e**). Sharpness of bronchi walls (arrowhead) and overall image quality were improved as the FOV was reduced from 320 to 240 or 160 mm. However, when the FOV was reduced from 160 to 80 or 40 mm, sharpness of bronchi walls (arrowhead) and overall image quality improved only slightly or were essentially unchanged

**Fig. 2:**
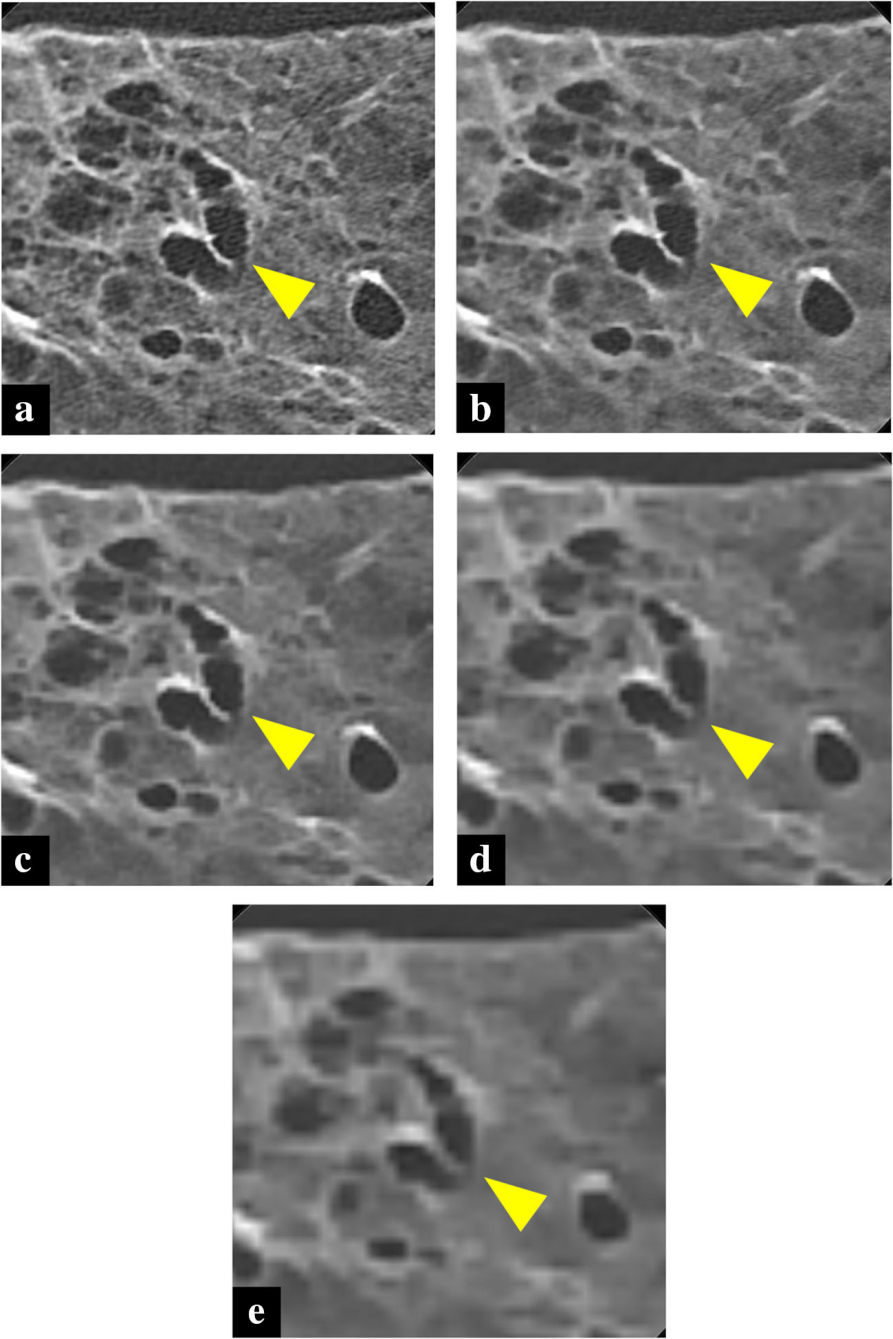
Images of U-HRCT of cadaveric lung (usual interstitial pneumonia) at FOV 40 mm (**a**), 80 mm (**b**), 160 mm (**c**), 240 mm (**d**), and 320 mm (**e**). Sharpness of bronchi walls (arrowhead) and overall image quality were improved as the FOV was reduced from 320 to 240, 160, or 80 mm. However, when the FOV was reduced from 80 to 40 mm, sharpness of bronchi walls (arrowhead) and overall image improved only slightly

The lungs were scanned with U-HRCT and conventional HRCT. U-HRCT images were obtained with a gantry rotation of 1.5 s, pitch factor 0.81, 120 kV, 200 mA, and CT dose index 23.2 mGy. Conventional HRCT images were obtained with a gantry rotation of 1.5 s, pitch factor 0.81, 120 kV, 200 mA, and CT dose index 23.9 mGy. Each lung was scanned at three different position levels. CT images with slice thickness 0.5 mm were reconstructed using the following FOV sizes: 40, 80, 160, 240, and 320 mm. The images were reconstructed at a 512 × 512 matrix size. FC81 was used as the reconstruction kernel (Figs. 1 and 2). In total, we obtained 330 images (66 per each FOV size).

### Evaluation of image quality

To clarify the influence of FOV in conventional HRCT and U-HRCT, three image quality evaluations were performed. In the first evaluation, conventional HRCT image quality was compared among the FOV values. In the second evaluation, the same comparison was performed using the U-HRCT images. In the third evaluation, the image quality of conventional HRCT was compared with that of U-HRCT. Image quality was evaluated using an 8.3-megapixel, 32-in. color liquid crystal display monitor.

### Evaluation of conventional HRCT image quality

In the first evaluation, we compared the image quality with conventional HRCT among the FOVs (40, 80, 160, 240, and 320 mm) in the following items: bronchi (*n* = 66), small vessel (*n* = 66), bronchiectasis (*n* = 6), bronchovascular bundle thickening (*n* = 4), consolidation (*n* = 4), emphysema (*n* = 5), faint nodules (*n* = 9), ground-glass opacity (*n* = 6), honeycombing (*n* = 9), interlobular septal thickening (*n* = 7), reticulation (*n* = 6), and solid nodules (*n* = 14). These items were marked in advance to obtain uniform evaluation points. Overall image quality (*n* = 66) was evaluated on the whole image. An image was randomly selected from the five FOVs and evaluated with respect to the image with FOV 160 mm at the same level, independently by three radiologists (with 8, 17, and 25 years of experience) on a 5-point scale. The images were presented in random order and the evaluators were blinded to the next image to be presented.

The scoring scale was defined in comparison to the reference image as follows: score 1, obviously poor image quality (almost impossible to detect structures or very difficult to clearly evaluate their margin or internal characteristics); score 2, poor image quality (possible to detect structures but difficult to clearly evaluate their margin or internal characteristics); score 3, fair image quality (margin or internal characteristics can be detected and evaluated as well as in the reference image); score 4, better image quality (easier to detect structures and to evaluate their margin or internal characteristics); and score 5, excellent image quality (significantly easier to detect structures and to evaluate their margin or internal characteristics, without any indistinct findings).

### Evaluation of U-HRCT image quality

In the second evaluation, the same comparisons were performed on U-HRCT images, using the same 5-point scale, by the same three radiologists in a blinded manner.

### Comparison of conventional HRCT and U-HRCT

In the third evaluation, we selected one image obtained with conventional HRCT and the U-HRCT image of the same case, at the same FOV and slice level, and evaluated overall image quality. The two images were displayed side by side, with random assignment of the side and the order of image presentation. The image presented on the right side was evaluated with reference to the image presented on the left side. Overall image quality was evaluated on the whole image. The scoring was independently performed by three radiologists in comparison with the reference image using a 3-point scale: score 1, poor image quality (possible to detect structures but difficult to clearly evaluate their margin or internal characteristics); score 2, fair image quality (margin or internal characteristics can be detected and evaluated as well as in the reference image); and score 3, excellent image quality (easier to detect structures and to evaluate their margin or internal characteristics, without any indistinct findings).

### Quantitative evaluation of noise

Image noise was quantitatively obtained using the workstation by measuring the standard deviation (SD) values of circular regions of interest (ROI), which were drawn on the air portion of images. We used the same monitor as in the abovementioned evaluations. It has been reported that noise can be quantitatively evaluated by measuring the SD value with an ROI in the air portion [11–13]. Images for which ROIs could be drawn with all FOVs were extracted, and one to three ROIs of the same size were placed at the same location on the selected conventional HRCT image and the U-HRTC image. The SD value was calculated in cases with one ROI, and the average SD in cases with multiple ROIs.

### Statistical analysis

In the first and second evaluation, the median values of the subjective scores of the three radiologists and the statistical significance of the differences among FOVs were analyzed using Shapiro–Wilk test to test normality and ANOVA with Bonferroni post hoc test. *P* values < 0.005 were considered significant. In the third evaluation, the median values of the subjective scores of the three radiologists and the statistical significance of the differences between conventional HRCT and U-HRCT at the same FOV were analyzed using Shapiro–Wilk test to test normality and two-way ANOVA with Bonferroni post hoc test. *P* values < 0.05 were considered significant. In the quantitative noise evaluation, two-way ANOVA and Bonferroni post hoc test were used to compare the various FOVs in each CT model (*p* < 0.005 considered significant) and the CT models at each FOV (*p* < 0.05 considered significant). In all evaluations, the results of normality test were nonparametric. SPSS (version 24; IBM) was used for statistical processing.

## Results

### Evaluation of conventional HRCT image quality

The scores (FOV 40, 80, 160, 240, and 320 mm) and the results of the comparisons between FOVs (40 vs. 80 mm, 40 vs. 160 mm, and 80 vs. 160 mm) are summarized in Table 1, while the results of the other comparisons are summarized in Supplemental Table 1 (*F* > 27.2, *p* < 0.001 in all ANOVA comparisons).

**Table 1:** Subjective evaluation of conventional HRCT: CT findings

	Score (mean ± SD)				
FOV size (mm)	40	80	160	240	320
Bronchi	3.5 ± 0.5*^,#^	3.3 ± 0.4^☨^	3.0 ± 0.0	1.6 ± 0.5	1.0 ± 0.0
Small vessel	3.4 ± 0.5^#^	3.2 ± 0.4	3.0 ± 0.0	1.7 ± 0.4	1.0 ± 0.0
Bronchiectasis	3.3 ± 0.5	3.2 ± 0.4	3.0 ± 0.0	1.8 ± 0.4	1.0 ± 0.0
Bronchovascular bundle thickening	3.3 ± 0.4	3.0 ± 0.0	3.0 ± 0.0	2.0 ± 0.0	1.0 ± 0.0
Consolidation	3.8 ± 0.4	3.3 ± 0.4	3.0 ± 0.0	2.0 ± 0.0	1.3 ± 0.4
Emphysema	3.0 ± 0.0	3.0 ± 0.0	3.0 ± 0.0	2.0 ± 0.0	1.0 ± 0.0
Faint nodule	3.4 ± 0.5	3.1 ± 0.3	3.0 ± 0.0	1.9 ± 0.3	1.0 ± 0.0
Ground-glass opacity	3.7 ± 0.5	3.2 ± 0.4	3.0 ± 0.0	1.8 ± 0.4	1.0 ± 0.0
Honeycombing	3.2 ± 0.3	3.1 ± 0.4	3.0 ± 0.0	1.8 ± 0.4	1.0 ± 0.0
Interlobular septal thickening	3.7 ± 0.5^##^	3.3 ± 0.5	3.0 ± 0.0	2.0 ± 0.0	1.0 ± 0.0
Reticulation	3.8 ± 0.4^#^	3.7 ± 0.5	3.0 ± 0.0	2.0 ± 0.0	1.0 ± 0.0
Solid nodule	3.3 ± 0.5	3.2 ± 0.4	3.0 ± 0.0	2.0 ± 0.0	1.0 ± 0.0
Overall image quality	3.3 ± 0.5**^,#^	3.1 ± 0.3	3.0 ± 0.0	1.9 ± 0.3	1.0 ± 0.0

Data are presented as mean ± SD. Subjective image analysis data were analyzed using Shapiro–Wilk test to test normality and ANOVA and Bonferroni post hoc tests. *p* values < 0.005 were considered significant. The scores of the evaluation items increased at a constant rate when the FOV was reduced to 160 mm, but only slightly when the FOV was further reduced

^*^ Significant difference between FOV 40 mm and FOV 80 mm (*p* = 0.004); ^**^ significant difference between FOV 40 mm and FOV 80 mm (*p* < 0.001); ^#^ significant difference between FOV 40 mm and FOV 160 mm (*p* < 0.001); ^##^ significant difference between FOV 40 mm and FOV 160 mm (*p* = 0.002); ^☨^ significant difference between FOV 80 mm and FOV 160 mm (*p* = 0.002)

Overall image quality scores clearly improved as the FOV was reduced from 320 to 240 or 160 mm (all *p* < 0.001). However, when the FOV was reduced from 160 to 80 mm, the score was not significantly different, and a further reduction from 80 to 40 mm led to a slight improvement (*p* = 0.002).

Likewise, the scores of all the evaluation items clearly improved as the FOV was reduced from 320 to 160 mm (*p* < 0.001). When the FOV was reduced from 160 to 80 or 40 mm, the scores improved only slightly (*p* < 0.001) or showed no significant differences.

### Evaluation of U-HRCT image quality

The scores for each FOV and the results of the comparison between the FOVs are summarized in Table 2, while the results of the other comparisons are shown in Supplemental Table 2 (*F* > 80.8, *p* < 0.001 in all ANOVA comparisons).

**Table 2:** Subjective evaluation of U-HRCT: CT findings

	Score (mean ± SD)				
FOV size (mm)	40	80	160	240	320
Bronchi	4.6 ± 0.5*^,#^	4.0 ± 0.0^☨^	3.0 ± 0.0	1.6 ± 0.5	1.0 ± 0.0
Small vessel	4.5 ± 0.5*^,#^	4.1 ± 0.3^☨^	3.0 ± 0.0	1.7 ± 0.4	1.0 ± 0.0
Bronchiectasis	4.5 ± 0.5^#^	4.0 ± 0.0^☨^	3.0 ± 0.0	1.8 ± 0.4	1.0 ± 0.0
Bronchovascular bundle thickening	4.3 ± 0.4^##^	4.3 ± 0.4^☨☨^	3.0 ± 0.0	2.0 ± 0.0	1.0 ± 0.0
Consolidation	5.0 ± 0.0^#^	4.5 ± 0.5^☨^	3.0 ± 0.0	2.0 ± 0.0	1.3 ± 0.4
Emphysema	4.6 ± 0.5^#^	4.2 ± 0.4^☨^	3.0 ± 0.0	2.0 ± 0.0	1.0 ± 0.0
Faint nodule	4.1 ± 0.6^#^	4.1 ± 0.3^☨^	3.0 ± 0.0	1.9 ± 0.3	1.0 ± 0.0
Ground-glass opacity	4.7 ± 0.5^#^	4.2 ± 0.4^☨^	3.0 ± 0.0	1.8 ± 0.4	1.0 ± 0.0
Honeycombing	4.3 ± 0.5^#^	4.0 ± 0.0^☨^	3.0 ± 0.0	1.8 ± 0.4	1.0 ± 0.0
Interlobular septal thickening	4.6 ± 0.5**^,#^	4.0 ± 0.0^☨^	3.0 ± 0.0	2.0 ± 0.0	1.0 ± 0.0
Reticulation	4.7 ± 0.5^#^	4.2 ± 0.4^☨^	3.0 ± 0.0	2.0 ± 0.0	1.0 ± 0.0
Solid nodule	4.3 ± 0.5^#^	4.0 ± 0.0^☨^	3.0 ± 0.0	2.0 ± 0.0	1.0 ± 0.0
Overall image quality	4.5 ± 0.5*^,#^	4.0 ± 0.2^☨^	3.0 ± 0.0	1.9 ± 0.3	1.0 ± 0.0

Data are presented as mean ± SD. Subjective image analysis data were analyzed using Shapiro–Wilk test to test normality and ANOVA and Bonferroni post hoc tests. *p* values < 0.005 were considered significant. The scores of the evaluation items increased at a constant rate when the FOV was reduced to 80 mm, but only slightly when the FOV was further reduced

^*^ Significant difference between FOV 40 mm and FOV 80 mm (*p* < 0.001); ^**^ significant difference between FOV 40 mm and FOV 80 mm (*p* = 0.001); ^#^ significant differences between FOV 40 mm and FOV 160 mm (*p* < 0.001); ^##^ significant difference between FOV 40 mm and FOV 160 mm (*p* = 0.001); ^☨^ significant difference between FOV 80 mm and FOV 160 mm (*p* < 0.001); ^☨☨^ significant difference between FOV 80 mm and FOV 160 mm (*p* = 0.001)

The overall image quality scores clearly improved as the FOV was reduced from 320 to 240, 160, or 80 mm (*p* < 0.001). When the FOV was reduced from 80 to 40 mm, the score improved slightly (*p* < 0.001).

Similarly, the scores of all the evaluations clearly improved as the FOV was reduced from 320 to 80 mm (*p* < 0.001). However, when the FOV was further reduced from 80 to 40 mm, the scores slightly improved (*p* < 0.001) or showed no significant differences.

### Comparison of conventional HRCT and U-HRCT

**Fig. 3:**
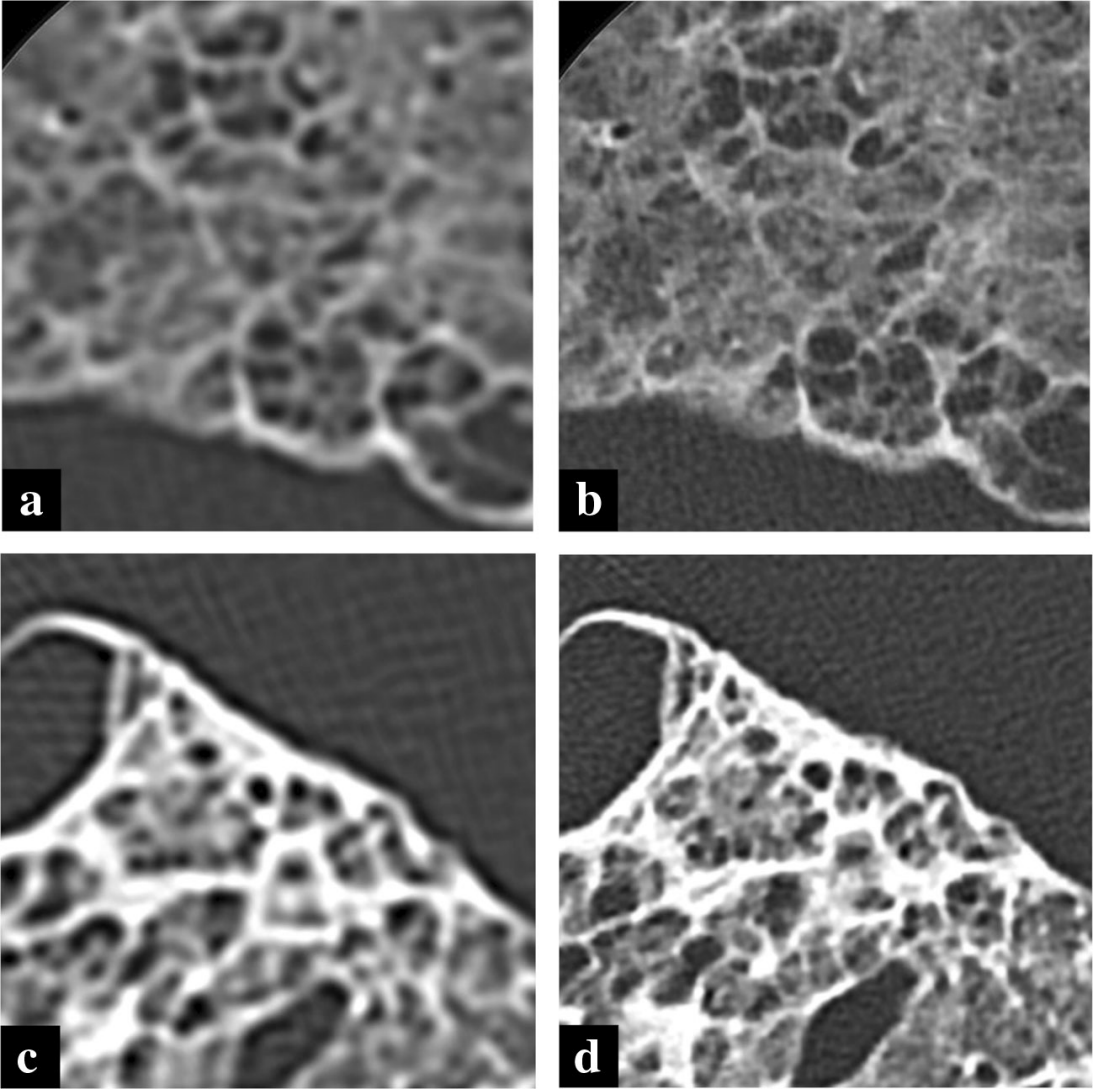
Images of conventional HRCT (**a**, **c**) and U-HRCT (**b**, **d**) of cadaveric lung (usual interstitial pneumonia) at FOV 40 mm. Overall image quality of U-HRCT was improved compared with conventional HRCT and noise was more severe in U-HRCT compared with conventional HRCT

**Fig. 4:**
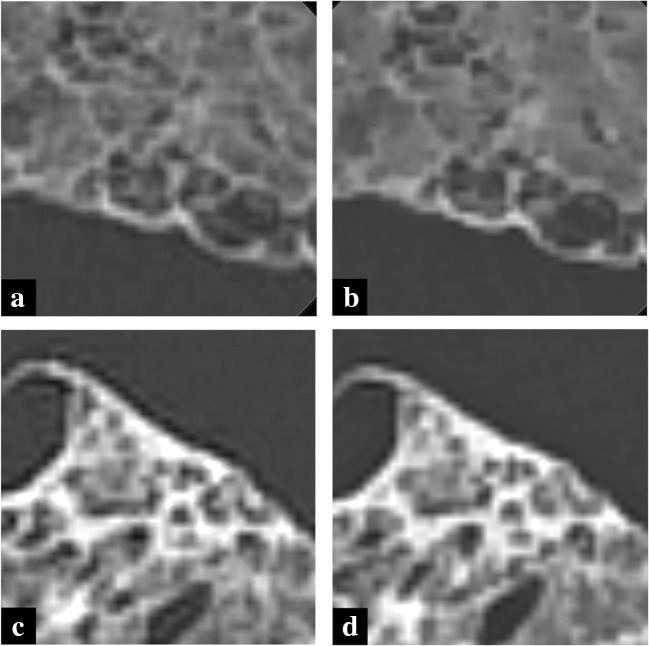
Images of conventional HRCT (**a**, **c**) and U-HRCT (**b**, **d**) of cadaveric lung (usual interstitial pneumonia) at FOV 320 mm. Overall image quality was improved in U-HRCT compared with conventional HRCT and no difference in noise was detected

The results are summarized in Table 3 (two-way ANOVA for the comparison among FOV sizes: *F* = 0.041, *p* = 0.997; two-way ANOVA analyzing CT model, FOV size, and their interaction: *F* > 5.6, *p* < 0.001). The U-HRCT overall image quality scores were superior to those of conventional HRCT at all FOVs (all *p* < 0.001, Figs. 3 and 4). A significant interaction between FOV size and CT model (*p* < 0.001) and a significant main effect of CT model (*p* < 0.001), but not of FOV (*p* = 0.997), were detected.

**Table 3:** Subjective comparison of U-HRCT and conventional HRCT: CT findings

	Score (mean ± SD)					
FOV size (mm)		40	80	160	240	320
Overall image quality	U-HRCT	3.0 ± 0.0*	3.0 ± 0.0*	3.0 ± 0.0*	3.0 ± 0.0*	2.9 ± 0.4*
	C-HRCT	1.0 ± 0.0	1.0 ± 0.0	1.0 ± 0.0	1.0 ± 0.0	1.1 ± 0.4

Data are presented as mean ± SD. The scores of the subjective overall image quality analysis were analyzed using Shapiro–Wilk test to test normality and two-way ANOVA and Bonferroni post hoc tests comparing conventional HRCT and U-HRCT at the same FOV. *P* values < 0.005 were considered significant

*Significant difference between U-HRCT and conventional HRCT (C-HRCT) (*p* < 0.001)

### Quantitative evaluation of noise

The results are summarized in Table 4 (two-way ANOVA including FOV size, CT model, and their interaction: *F* > 235, *p* < 0.001). In conventional HRCT, there were significant differences between all pairs of FOVs (*p* < 0.001), except between 40 and 80 mm (*p* = 0.13). In U-HRCT, there were significant differences between all pairs of FOVs (*p* < 0.001). When comparing conventional HRCT with U-HRCT at each FOV, there were significant differences at FOV 40, 80, and 160 mm (*p* < 0.001), but not at 240 and 320 mm (*p* = 0.12 and *p* = 0.13, respectively). A significant interaction between FOV size and CT model was detected (*p* < 0.001), as well as significant main effects of both FOV size and CT model (both *p* < 0.001).

**Table 4:** Quantitative evaluation of noise on conventional HRCT and U-HRCT: SD value

	Score (mean ± SD)					
FOV size (mm)		40	80	160	240	320
Noise	U-HRCT	42.0 ± 6.4*	29.7 ± 4.2*	17.8 ± 1.7*	9.4 ± 0.9	5.9 ± 0.6
	C-HRCT	20.4 ± 6.5	19.2 ± 6.9	15.3 ± 4.8	10.2 ± 3.1	6.7 ± 1.7

The SD values are presented as mean ± SD. Quantitative noise evaluation data were analyzed using two-way ANOVA and Bonferroni post hoc tests. *p* values < 0.005 were considered significant. In U-HRCT, there were significant differences among all FOVs (*p* < 0.001). In conventional HRCT, all differences were significant except that between FOV 40 and 80 mm (*p* = 0.13)

*At FOV 40, 80, and 160 mm, there were significant differences between conventional HRCT and U-HRCT (*p* < 0.001)

## Discussion

Conventional HRCT image quality showed significant improvements when the FOV was reduced to 160 mm, but further FOV reduction led to slight or nonsignificant improvements. Similar results were obtained for U-HRCT; however, significant improvements could be observed when the FOV was reduced to 80 mm. Comparing U-HRCT and conventional HRCT, the U-HRCT overall image quality scores were superior to those of conventional HRCT at all FOVs. The image noise of U-HRCT, but not conventional HRCT, was increased when the FOV was reduced from 80 to 40 mm.

The spatial resolution of conventional HRCT is in the range of 0.23–0.35 mm [5, 9, 10]. When using a 512 × 512 matrix size, the size of one pixel at FOV 160 mm (80 mm) is 0.313 mm (0.156 mm). Therefore, the spatial resolution of conventional HRCT is higher than the pixel size at FOV 160 mm and lower at FOV 80 mm. These considerations are relevant to the quantitative evaluation of noise. It has been reported that image quality is improved and the noise is enhanced at higher resolution [3, 4]. We can conclude that in conventional HRCT improvements in image quality cannot be expected, and noise would not significantly increase, when the FOV is reduced from 80 to 40 mm, because the pixel size becomes smaller than the resolution. In U-HRCT, the spatial resolution is 0.14 mm [5, 7–9]. When using a 512 × 512 matrix size, the pixel size at FOV 40 mm is 0.078 mm. Therefore, the spatial resolution of U-HRCT is higher (lower) than the pixel size at FOV 80 mm (40 mm). Therefore, improvements in U-HRCT image quality can be expected as the FOV is reduced to 80 mm, because the resolution of U-HRCT is still higher than the pixel size, and the same applies to image noise. The quantitative evaluation of noise showed a synergic effect of FOV size and CT model, and both factors affected image noise independently. In particular, U-HRCT noise becomes more severe compared with conventional HRCT when the FOV is reduced. At FOV 240 and 320 mm, noise is almost equal in U-HRCT and HRCT, suggesting that the noise increase associated with U-HRCT might not affect clinical practice, where these or larger FOVs are frequently used.

Therefore, the use of U-HRCT allows clearer diagnostic images compared with conventional HRCT, without increasing noise. A study reported that in U-HRCT, minute structures such as bronchi were clearly delineated, and noise was reduced, in comparison with conventional HRCT [14]. It was also reported that images acquired using U-HRCT with a 0.25-mm slice thickness showed improved performance compared with a 0.5-mm thickness in visualizing the Adamkiewicz artery [15] and allowed visualizing calcified lesions of the coronary artery with fewer artifacts [16]. These reports support the results of our study. Regarding structures such as nodules, the average interslice volume was reported to be reduced, and small nodule detection improved, by using thin sections [17].

Image quality affects the quantitative and qualitative evaluation of lung field lesions [18, 19]. Improvement in image quality in the lung field plays an important role in diagnosis. Studies reported that U-HRCT can improve nodule images in the lung or bronchi, thereby improving diagnostic confidence [6, 20]. It was also reported that when using U-HRCT with a small FOV (100 mm), the lung nodule image quality and diagnostic confidence were improved compared with conventional HRCT with a larger FOV (350 mm) [18]. In summary, U-HRCT can be expected to improve the quality of diagnostic imaging of the lung and reduce the noise in clinical practice by allowing reduced FOVs.

This study has some limitations. Cadaveric lungs were used, and we did not evaluate the effects of absorption and scattering in the thorax on image quality. Moreover, image evaluation using cadaveric lungs does not include respiratory variation or fluctuations of the chest wall. Thus, image acquisition in this study does not necessarily correspond to actual clinical practice, making unclear to what extent our results can be applied to clinical practice; it will be necessary to study the influence of these fluctuations on U-HRCT image quality. Moreover, the sample size was relatively small. Regarding the evaluation procedure, the radiologists who evaluated the various items may have been unconsciously influenced by other information such as noise. Thus, it is possible that the evaluations of the different items were not completely independent. The side-by-side comparison used to evaluate conventional HRCT and U-HRCT may bias the radiologist’s evaluation. Only Canon CT scanners were used in the study, and therefore, strictly speaking, the results can be applied to U-HRCT developed by Canon Medical Systems, and it is unknown whether the same results would apply to U-HRCT developed by other companies. Despite our best efforts to match the slice position, the match was not perfect, so that the slice positions of the evaluation image and the reference image were slightly different, and such slight difference in slice position may have affected the image quality evaluation. It was reported that the FC81 reconstruction kernel performs different processes between conventional HRCT and U-HRCT [14], and such difference in reconstruction may affect image quality. We used pathologically diagnosed cadaveric lungs to our study, but we did not correlate abnormal CT findings such as solid nodules with the pathological assessments. The cadaveric lungs were inflated and fixed using the Heitzman’s method, and we do not know in detail the effects of such procedure on lung tissue, which could in principle affect both pathological diagnosis and image evaluation. However, the conventional HRCT and U-HRCT images were acquired almost at the same time, so that there was no difference in the quality of the cadaveric lungs between conventional HRCT and U-HRCT. A method to evaluate image quality by measuring tumor SNR has been reported [21], but SNR was not measured in this study. Because some evaluation items were not uniform (such as honeycombing, reticulation, etc.), unlike in tumors, when setting ROIs, the signal varied with the evaluation items, leading to instability of the quantitative evaluation.

In conclusion, since the spatial resolution of U-HRCT is higher than that of conventional HRCT, the limit of FOV at which image quality improves with conventional HRCT is 160 mm, while with U-HRCT image quality improves even when the FOV is reduced to 80 mm. Moreover, at all FOVs, U-HRCT image quality is improved compared with conventional HRCT. Thus, improvement of clinical diagnostic performance may be expected using U-HRCT, although further clinical studies will be needed.

## Electronic supplementary material

ESM 1 Supplemental Fig. 1 A graph of the overall image quality median scores of conventional HRCT. Bar graphs represent the scores and error bars represent standard deviations. Differences between all FOVs were significant with the exception of 160 vs 80 mm. Overall image quality was strongly improved when comparing a FOV of 320 mm with 240 mm, 160 mm, 80 mm, and 40 mm, and a FOV of 240 mm with 160 mm, 80 mm, and 40 mm. Overall image quality was slightly improved when comparing a FOV of 160 mm with 40 mm, and 80 mm with 40 mm (*p* < 0.001). Supplemental Fig. 2 Graph of the overall image quality scores of U-HRCT. The bar graphs represent the scores and error bars represent standard deviation. Differences among all FOVs were significant. Overall image quality was strongly improved when comparing a FOV of 320 mm with 240 mm, 160 mm, 80 mm, and 40 mm, a FOV of 240 mm with 160 mm, 80 mm, and 40 mm, and a FOV of 160 mm with 80 mm and 40 mm. Overall image quality was slightly improved when comparing a FOV of 80 mm with 40 mm (*p* < 0.001). Supplemental Fig. 3 A graph of the overall image quality scores of U-HRCT (left bar) and conventional HRCT (right bar). The bar graphs represent the scores and error bars represent standard deviations. U-HRCT image quality was significantly higher than that of conventional HRCT at all FOVs (*p* < 0.001). (DOCX 528 kb)

## Abbreviations

Conventional HRCT: Conventional high-resolution computed tomography
CT: Computed tomography
CT dose index: Computed tomography dose index
FOV: Field of view
HRCT: High-resolution CT
ROI: Region of interest
SD: Standard deviation
SNR: Signal-to-noise ratio
U-HRCT: Ultra-high-resolution computed tomography

## References

[1] Seemann MD, Staebler A, Beinert T (1999). Usefulness of morphological characteristics for the differentiation of benign from malignant solitary pulmonary lesions using HRCT. Eur Radiol.

[2] Zwirewich CV, Vedal S, Miller RR, Muller NL (1991). Solitary pulmonary nodule: high-resolution CT and radiologic–pathologic correlation. Radiology.

[3] Barrett JF, Keat N (2004). Artifacts in CT: recognition and avoidance. Radiographics.

[4] Boas FE, Fleischmann D (2012). CT artifacts: causes and reduction techniques. Imaging Med.

[5] Kakinuma R, Moriyama N, Muramatsu Y (2015). Ultra-high-resolution computed tomography of the lung: image quality of a prototype scanner. PLoS One.

[6] Zhu H, Zhang L, Wang Y (2017). Improved image quality and diagnostic potential using ultra-high-resolution computed tomography of the lung with small scan FOV: a prospective study. PLoS One.

[7] Hata A, Yanagawa M, Honda O (2018). Effect of matrix size on the image quality of ultra-high-resolution CT of the lung: comparison of 512 × 512, 1024 × 1024, and 2048 × 2048. Acad Radiol.

[8] Yanagawa M, Tomiyama N, Honda O (2010). Multidetector CT of the lung: image quality with garnet-based detectors. Radiology.

[9] Tsukagoshi S, Ota T, Fujii M, Kazama M, Okumura M, Johkoh T (2007). Improvement of spatial resolution in the longitudinal direction for isotropic imaging in helical CT. Phys Med Biol.

[10] Markarian B, Dailey ET, Groskin SA (1993). Preparation of inflated lung specimens. Heitzman’s the lung: radiologic-pathologic correlations.

[11] Boehm T, Willmann JK, Hilfiker PR (2003). Thin-section CT of the lung: dose electrocardiographic triggering influence diagnosis?. Radiology.

[12] Baumueller S, Winklehner A, Karlo C (2012). Low-dose CT of the lung: potential value of iterative reconstructions. Eur Radiol.

[13] Booij R, Dijkshoorn ML, van Straten M (2017). Efficacy of a dynamic collimator for overranging dose reduction in a second- and third-generation dual source CT scanner. Eur Radiol.

[14] Yanagawa M, Hata A, Honda O (2018). Subjective and objective comparisons of image quality between ultra-high-resolution CT and conventional area detector CT in phantoms and cadaveric human lungs. Eur Radiol.

[15] Yoshioka K, Tanaka R, Takagi H (2018). Ultra-high-resolution CT angiography of the artery of Adamkiewicz: a feasibility study. Neuroradiology.

[16] Motoyama S, Ito H, Sarai M (2018). Ultra-high-resolution computed tomography angiography for assessment of coronary artery stenosis. Circ J.

[17] Fischbach F, Knollmann F, Griesshaber V, Freund T, Akkol E, Felix R (2003). Detection of pulmonary nodules by multislice computed tomography: improved detection rate with reduced slice thickness. Eur Radiol.

[18] Goo JM, Tongdee T, Tongdee R, Yeo K, Hildebolt CF, Bae KT (2005). Volumetric measurement of synthetic lung nodules with multi-detector row CT: effect of various image reconstruction parameters and segmentation thresholds on measurement accuracy. Radiology.

[19] Zerhouni EA, Spivey JF, Morgan RH, Leo FP, Stitik FP, Siegelman SS (1982). Factors influencing quantitative CT measurements of solitary pulmonary nodules. J Comput Assist Tomogr.

[20] Sheshadri A, Rodriguez A, Chen R (2015). Effect of reducing field of view on multidetector quantitative computed tomography parameters of airway wall thickness in asthma. J Comput Assist Tomogr.

[21] Beer L, Toepker M, Ba-Ssalamah A (2019). Objective and subjective comparison of virtual monoenergetic vs. polychromatic images in patients with pancreatic ductal adenocarcinoma. Eur Radiol.

