# Supplementary material for: Influence of field of view size on image quality: ultra-high-resolution CT vs. conventional high-resolution CT
Source: Eur Radiol. 2020 Feb 18;30(6):3324–33. doi: 10.1007/s00330-020-06704-0 (PMC7248011; doi:10.1007/s00330-020-06704-0)

**Supplementary Materials**

**Supplementary Figure 1**


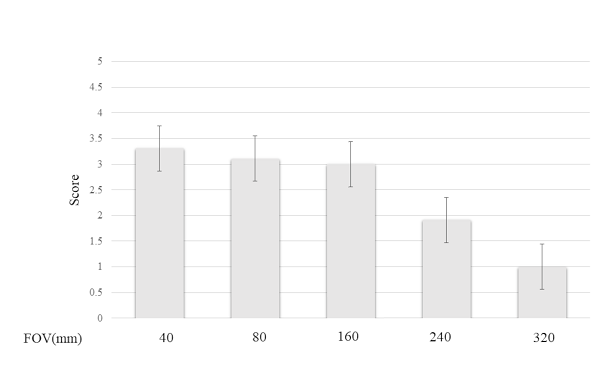


**Supplementary Figure 2**


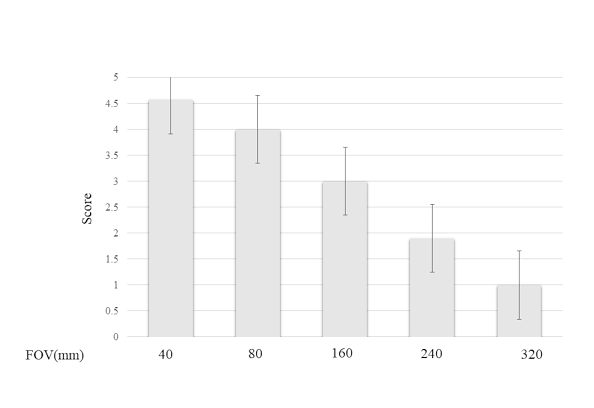


**Supplementary Figure 3**


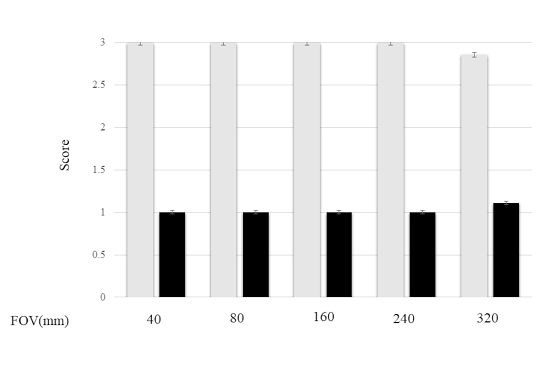


**Supplementary Table 1**


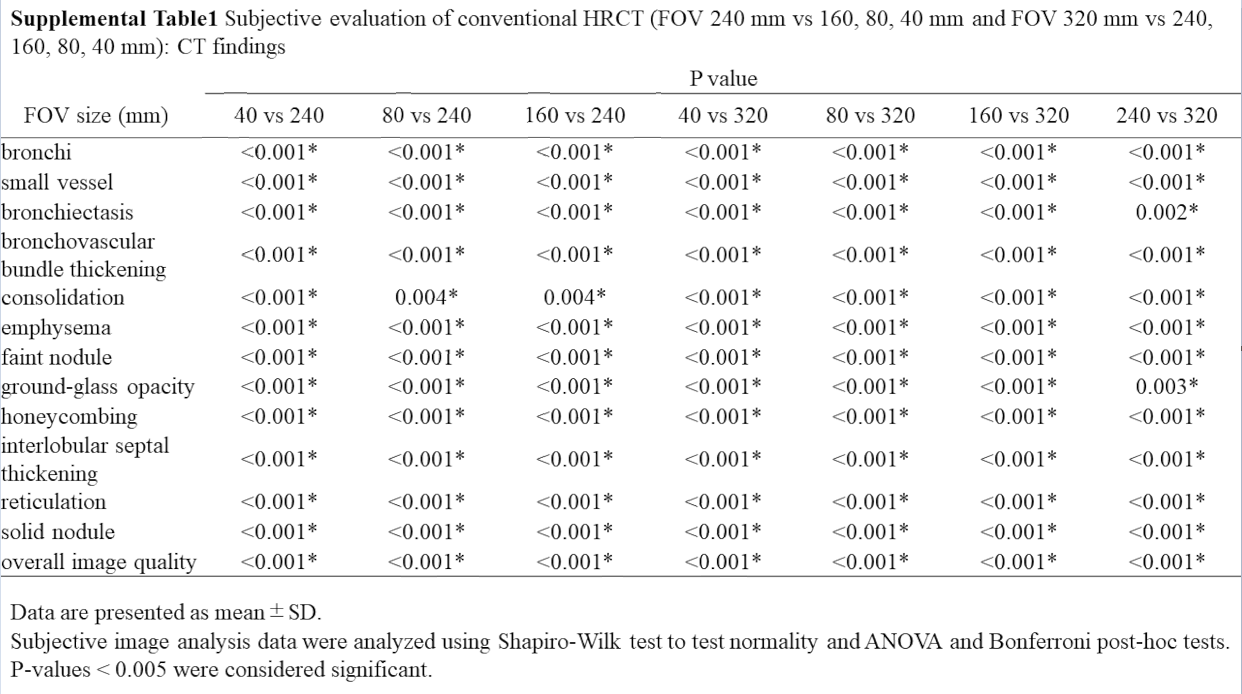


**Supplementary Table 2**


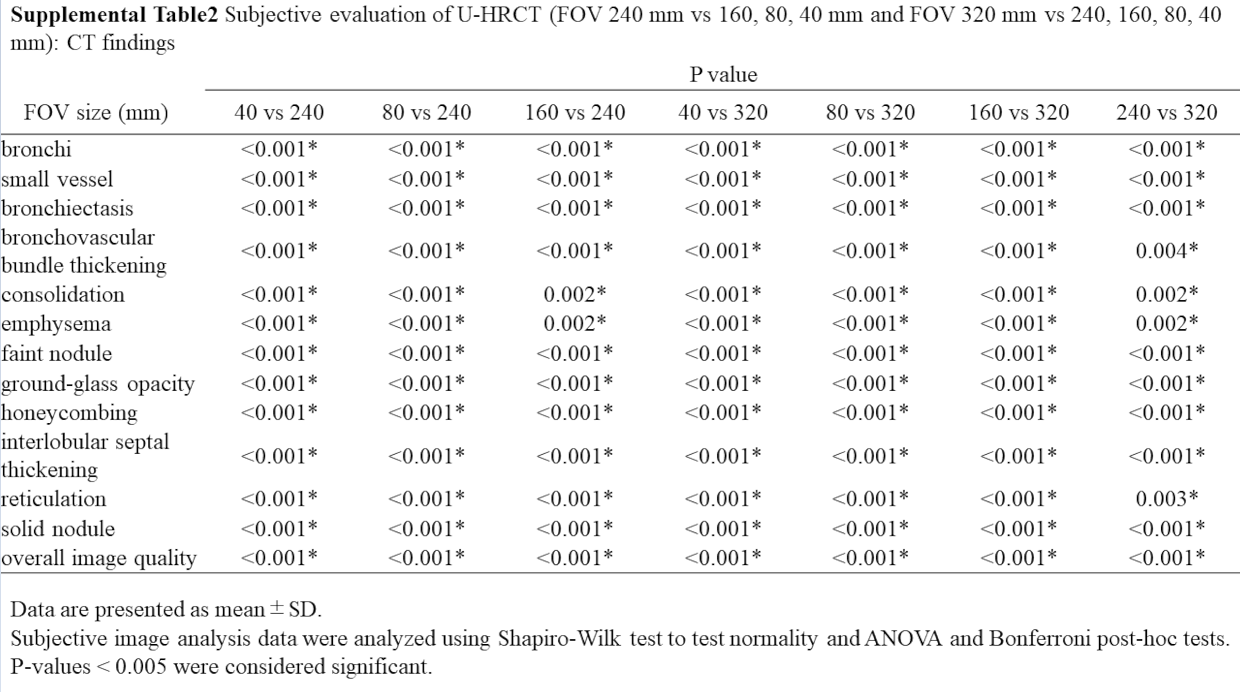

Supplement: Supplementary file 1 — Supplemental Fig. 1 A graph of the overall image quality median scores of conventional HRCT. Bar graphs represent the scores and error bars represent standard deviations. Differences between all FOVs were significant with the exception of 160 vs 80 mm. Overall image quality was strongly improved when comparing a FOV of 320 mm with 240 mm, 160 mm, 80 mm, and 40 mm, and a FOV of 240 mm with 160 mm, 80 mm, and 40 mm. Overall image quality was slightly improved when comparing a FOV of 160 mm with 40 mm, and 80 mm with 40 mm (p < 0.001). Supplemental Fig. 2 Graph of the overall image quality scores of U-HRCT. The bar graphs represent the scores and error bars represent standard deviation. Differences among all FOVs were significant. Overall image quality was strongly improved when comparing a FOV of 320 mm with 240 mm, 160 mm, 80 mm, and 40 mm, a FOV of 240 mm with 160 mm, 80 mm, and 40 mm, and a FOV of 160 mm with 80 mm and 40 mm. Overall image quality was slightly improved when comparing a FOV of 80 mm with 40 mm (p < 0.001). Supplemental Fig. 3 A graph of the overall image quality scores of U-HRCT (left bar) and conventional HRCT (right bar). The bar graphs represent the scores and error bars represent standard deviations. U-HRCT image quality was significantly higher than that of conventional HRCT at all FOVs (p < 0.001). (DOCX 528 kb) [file 330_2020_6704_MOESM1_ESM.docx]
